# E5564 inhibits immunosuppressive cytokine IL-10 induction promoted by HIV-1 Tat protein

**DOI:** 10.1186/s12985-014-0214-z

**Published:** 2014-12-04

**Authors:** Elmostafa Bahraoui, Laurence Briant, Nathalie Chazal

**Affiliations:** Université Paul Sabatier, EA 3038, 118 Route de Narbonne, 31062 Toulouse, France; INSERM, U1043, CPTP, CHU Purpan, BP3028, 31024 Toulouse, Cedex 3 France; CNRS, U5282, CPTP, CHU Purpan, BP3028, 31024 Toulouse, Cedex3 France; Centre d’études d’agents Pathogènes et Biotechnologies pour la Santé (CPBS), UMR5236, CNRS - Université Montpellier 1-Montpellier 2, Montpellier, France

**Keywords:** Tat, Monocyte, Macrophage, TLR4, IL-10, E5564

## Abstract

**Background:**

In HIV-1 infected patients, production of interleukin-10 (IL-10), a highly immunosuppressive cytokine, is associated with progression of infection toward AIDS. HIV-1 Tat protein, by interacting with TLR4-MD2 at the membrane level, induces IL-10 production by primary human monocytes and macrophages. In the present study we evaluated the effect of the TLR4 antagonist Eritoran tetrasodium (E5564) on HIV-1 Tat-induced IL-10 production.

**Findings:**

Here, we confirm that the recombinant HIV-1 Tat protein and the GST-Tat 1–45 fusion protein efficiently stimulate IL-10 production by primary monocytes and macrophages and that this stimulation is inhibited by blocking anti-TLR4 mAbs. We show that a similar inhibition is observed by preincubating the cells with the TLR4 antagonist E5564.

**Conclusion:**

This study provides compelling data showing for the first time that the TLR4 antagonist E5564 inhibits the immunosuppressive cytokine IL-10 production by primary human monocytes and macrophages incubated in the presence of HIV-1 Tat protein.

## Introduction

In HIV-1 infected patients, the deregulation of the immune system precedes the decline of the T CD4^+^ lymphocytes population. This immune disorder is mainly associated with the loss of T-cell proliferation in response to stimulating antigens and with a shift from Th1 to Th2 cytokines profile, leading to high levels of circulating TNF-α, IL-1, IL-4, IL-6, IL-12. This deregulation is accompanied by an enhanced secretion of IL-10 [1-5], an immunosuppressive cytokine crucial for the global immune dysfunction occurring during the course of HIV-1 infection [6]. Indeed, peripheral blood mononuclear cells (PBMCs) from HIV-1-positive patients produce high levels of IL-10, whose level increases as the patient progresses toward AIDS.

In addition to its role in viral gene expression, the HIV-1 Tat transactivating protein plays a key role in the dysregulation of the host immune system. Tat is secreted by infected cells and detected at the nM level in the serum of HIV-1 positive patients [7-9]. This cell-free protein exerts bystander effects on other cells whether or not they are infected, leading to the modulation of cellular genes expression. In this field, HIV-1 Tat, by acting at the cell membrane surface, stimulates IL-10 and TNF-α secretion by human monocytes and macrophages [10,11]. Different domains in the HIV-1 Tat protein have been implicated in interactions with various cell receptors: (1) the N-terminal region in Tat binds the CD26 receptor expressed at the lymphocyte cell membrane; (2) the tripeptide RGD (Arginine-Glycine-Aspartate) motif interacts with α_v_β_3_ and α_5_β_1_ integrins at the surface of dendritic cells; (3) the basic region recruits membrane lipids and the VEGF receptor expressed by endothelial cells [12]; (4), Albini *et al*. reported the interaction of the cysteine-rich region in Tat (24–51) with CCR2, CCR3 and CXCR4 chemokines receptors [13]. Due to this last property, Tat was proposed to compete with infection by X4-tropic HIV strains; (5). More recently, we reported that Tat N-terminal domain, by interacting with the Toll-like receptor 4-myeloid differentiation factor 2 complexes (TLR4-MD2), promotes TNF-α and IL-10 secretion by macrophages and monocytes [14]. Given the crucial importance of IL-10 in immune dysfunction and the capacity of IL-10 to synergize with inflammatory cytokines to enhance viral replication in HIV-1-positive patients, inhibition of Tat/TLR4-MD2 may represent in the long term an attractive therapeutic strategy.

TLR4 antagonists include molecules such as Eritoran tetrasodium (E5564) and its predecessors (E5531) [15], Resatorvid (TAK 242, a small molecule inhibitor of TLR4-CD14 mediated intracellular signaling), and antibodies targeting the TLR4 receptor. Of note, some therapeutic agents such as ketamine, opioids and statins may also non-selectively interfere with TLR4 [16-18]. E5564 is a structural analog of the A lipid from *R sphaeroides* (RsLA), originally synthesized at the Eisai Research Institute of Boston (Andover, MA) [19]. E5564 competitively binds to TLR4-MD2, prevents LPS-induced NF-κB activation, inhibits TNF-α, IL-1β, IL-6 and IL-10 release *in vitro* and *in vivo*, and abolishes inflammatory responses in animal and human models of endotoxemia [15] without significant intrinsic agonistic effects.

**Figure 1:**
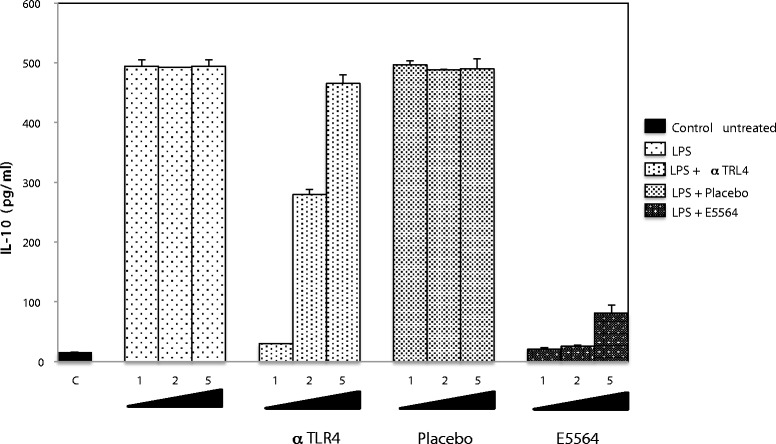
**E5564 inhibits LPS-induced production of IL-10 by primary human monocytes.** Primary human monocytes were treated or not by anti-TLR4 blocking antibodies (1 μg/ml), or with placebo (10 nM) or with E5564 (10 nM) before stimulation by increasing concentration of LPS (1, 2, 5 ng/ml). The data represent means concentrations of IL-10 in culture supernatants ± SD of three independent experiments.

According to these properties, the present study was designed to evaluate the capacity of E5564 to inhibit Tat-induced IL-10 production by human monocytes and macrophages. First, we determined the optimal monocytes culture conditions required for inhibition of LPS-induced secretion of IL-10 by E5564. PBMCs were isolated from buffy coats from HIV-negative donors by Ficoll density gradient centrifugation (Pharmacia). The cells were resuspended in 60/30 complete medium (60% AIM V and 30% Iscove (Gibco) containing penicillin (100 IU/ml), streptomycin (100 μg/ml) and 10% FCS and cultured for 24 h at 37°C in 5% CO_2_ (10^6^ cells/well) in 24-well Primaria (Becton Dickinson) tissue culture plates for 24 hours at 37°C in 5% CO_2_ in order to separate monocytes by plastic adherence. Non-adherent cells were removed, the remaining cells were washed twice and then stimulated by increasing concentrations of LPS either directly or after preincubation with 1 μg/ml HTA125 anti-TLR4 blocking monoclonal antibody (mAb) (eBioscience), or with 10 nM placebo or with 10 nM E5564 (placebo and E5564 were kindly provided by Eisai Research Institute of Boston). Used at these concentrations E5564 and placebo displayed no cytotoxic effect (data not shown) [20]. After 24 h in culture, supernatants were collected and analyzed for human IL-10 content (ELISA kit, BD Biosciences). In our hands, 10 nM E5564 were effective at reducing IL-10 production to background levels following stimulation with 1 to 2 ng/ml LPS. No inhibition was observed when LPS stimulation was performed in the presence of placebo (Figure 1). Notably, this inhibition was more efficiently achieved using the E5564 molecule than the anti-TLR4 mAbs.

**Figure 2:**
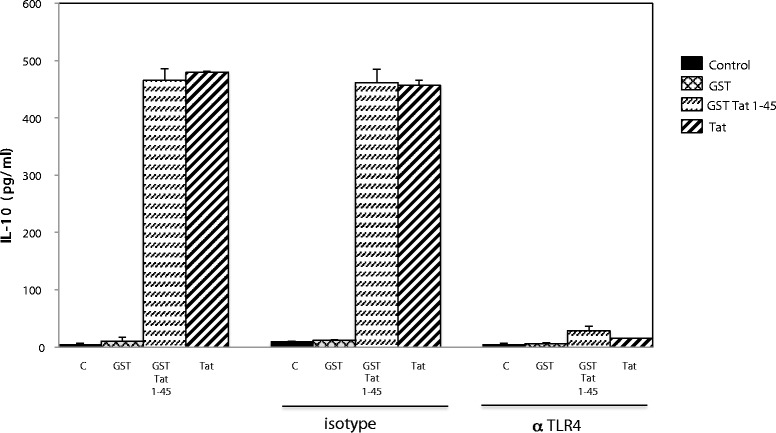
**HIV-1 Tat-induced IL-10 secretion by monocytes is TLR4-dependent.** Monocytes pre-incubated or not with blocking antibodies against TLR4 (1 μg/ml) or isotype-matched mAb were treated with HIV-1 Tat protein (10 nM), GST-Tat 1–45 (10 nM) or GST (10 nM) as control. After 24 h, IL-10 in the supernatant was measured by ELISA. The data represent means ± SD of three independent experiments.

Next, we determined *in vitro* effects of the HIV-1 Tat protein on IL-10 production by human monocytes. Recombinant HIV-1 Tat protein 1–86 (obtained from the Agence Nationale de la Recherche sur le SIDA, Paris, France) or recombinant GST-Tat 1–45 produced from our laboratory as previously described [21] and controlled for endotoxin contamination using the Limulus amebocyte lysate (LAL) assay (Bio-Sepra, France) [10,21-23] were added to primary human monocytes pre-incubated or not of with the HTA125 anti-TLR4 mAb or with a non-specific isotype-matched IgG (1 μg/ml). The supernatant was collected 24 h post-stimulation and analyzed for human IL-10 content as previously described [21]. Using this approach, we showed that stimulation with recombinant Tat protein or recombinant GST-Tat 1–45 equally stimulated IL-10 production (Figure 2). In contrast, we found that anti-TLR4 antibodies dramatically decreased both Tat and GST-Tat 1-45-induced cytokine release. No inhibition was observed when Tat or GST Tat-145 stimulation was performed in the presence of irrelevant isotype mAb (Figure 2).

**Figure 3:**
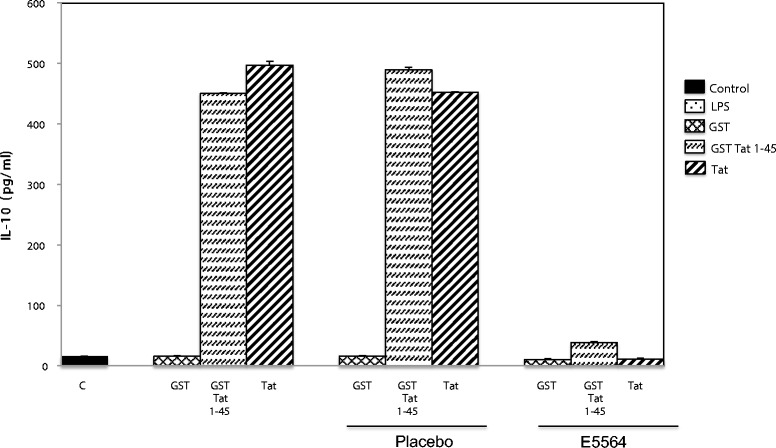
**E5564 counteracts HIV-1-Tat- and GST-Tat 1-45-induced secretion of IL-10 by human monocytes.** Primary human monocytes were preincubated or not with E5564 (10 nM) or placebo (10 nM) before stimulation by HIV-1 Tat protein (10 nM), GST-Tat 1–45 (10 nM) or GST (10 nM) as a control. The data represent means ± SD of three independent experiments.

**Figure 4:**
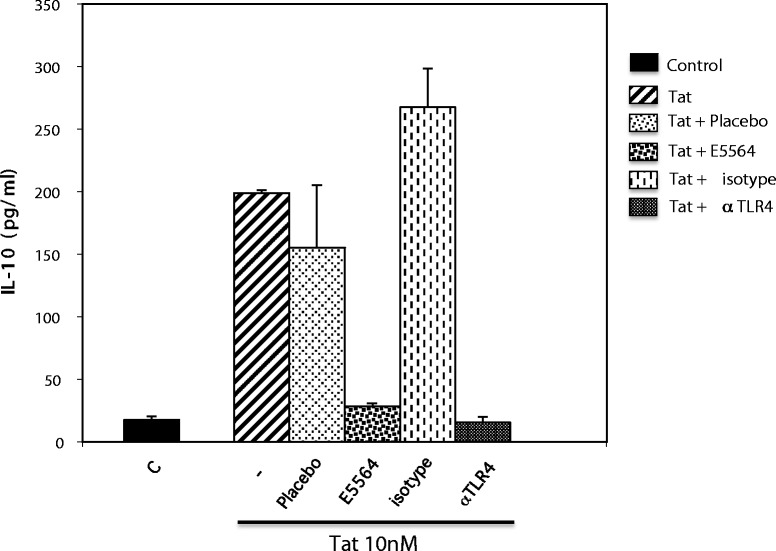
**E5564 inhibits the release of IL-10 immunosuppressive cytokine promoted by the HIV-1 Tat protein in primary human macrophages.** Primary human macrophages were left untreated or preincubated with anti-TLR4 blocking antibodies (1 μg/ml) or isotype-matched non-specific mAb or E5564 (10 nM) or placebo (10 nM) before stimulation by the HIV-1 Tat protein (10 nM). The data represent means ± SD of three independent experiments.

We next evaluated the consequences of E5564 treatment on IL-10 production by monocytes stimulated with recombinant Tat or GST-Tat 1–45. We found that 10 nM of E5564 were effective at counteracting the stimulating effects of recombinant Tat or recombinant GST-Tat 1–45. This effect was not observed when the cells were incubated with the same concentration of placebo (Figure 3). According to this observation, E5564 was effective at inhibiting Tat-induced IL-10 secretion by monocytes. Finally, these experiments were repeated using primary human macrophages as target cells. Monocytes prepared from PBMCs by plastic adhesion were differentiated into macrophages by incubation in a 10% FCS, 1% M-CSF and 1% PS mixture. Blood monocytes adhered to plastic after 1 h and acquired macrophage-like morphology within 5 days. On day 7, differentiated macrophages were stimulated with the recombinant HIV-1 Tat protein in presence of anti-TLR4 mAb, or irrevelant isotype mAb, or placebo or E5564. In these conditions E5564 and anti-TLR4 mAb inhibited Tat-induced cytokine production. No inhibition was observed when macrophages were incubated with the placebo molecule or with the isotype-matched non-specific mAb (Figure 4).

Altogether, these results indicate that the TLR4 agonist E5564 inhibits Tat-induced secretion of IL-10, by primary human monocytes and macrophages. This molecule was recently shown to represent a novel issue in therapeutic management of inflammation associated with influenza infection [24-26] and treatment for sepsis [27]. The powerful immunosuppressive properties of IL-10, the strong association between elevated serum concentrations of this immunosuppressive Th2 cytokine with disease progression in HIV-1-infected patients together with the capacity of the retroviral Tat protein to stimulate IL-10 release through TLR4 binding strongly supports that inhibition of Tat/TLR4-MD2 interactions may represent a good candidate to decrypt the mechanisms responsible for IL-10 deregulation in HIV infection. In this respect, E5564 represents an attractive tool for understanding how HIV infection induces a state of immunodeficiency.

### Statistical tests

All statistical analyses used the Student’s t-test, unpaired for normal distribution, for at least three independent experiments. Differences were considered significant at p values < 0.05. Microsoft Excel and Prism were used to construct the plots and measure means, standard deviations and p values.

## Abbreviations

PBMCs: Peripheral blood mononuclear cells
IL-10: interleukin-10
TLR-4: Toll-like receptor 4
LPS: Lipopolysaccharide
E5564: Eritoran tetrasodium
GST: Glutathione S-transferase
